# Does regional implementation of a clinical pathway for older adult patients with pelvic fragility fractures after low-energy trauma improve patient outcomes (PELVIC): a multicentre, stepped-wedge, randomised controlled trial

**DOI:** 10.1136/bmjopen-2023-083809

**Published:** 2024-08-13

**Authors:** Anna H M Mennen, Marte Lommerse, Robert Hemke, Hanna C Willems, Mario Maas, Frank W Bloemers, Kees Jan Ponsen, Daphne Van Embden

**Affiliations:** 1 Department of Surgery, Amsterdam UMC Location AMC, Amsterdam, The Netherlands; 2 Department of Radiology and Nuclear Medicine, Amsterdam UMC Location AMC, Amsterdam, The Netherlands; 3 Department of Internal Medicine and Geriatrics, Amsterdam UMC Locatie AMC, Amsterdam, The Netherlands; 4 Department of Surgery, Amsterdam UMC Location VUmc, Amsterdam, The Netherlands; 5 Department of Surgery, Noordwest Ziekenhuisgroep, Alkmaar, The Netherlands

**Keywords:** aged, orthopaedic & trauma surgery, adult orthopaedics

## Abstract

**Introduction:**

Patients with pelvic fragility fractures suffer from high morbidity and mortality rates. Despite the high incidence, there is currently no regional or nationwide treatment protocol which results in a wide variety of clinical practices. Recently, there have been new insights into treatment strategies, such as early diagnosis and minimally invasive operative treatment. The aim of this study is to implement an evidence-based and experience-based treatment clinical pathway to improve outcomes in this fragile patient population.

**Methods and analysis:**

This study will be a regional stepped-wedge cluster randomised controlled trial. All older adult patients (≥50 years old) who suffered a pelvic fragility fracture after low-energetic trauma are eligible for inclusion. The pathway aims to optimise the diagnostic process, to guide the decision-making process for further treatment (eg, operative or conservative), to structure the follow-up and to provide guidelines on pain management, weight-bearing and osteoporosis workup. The primary outcome is mobility, measured by the Parker Mobility Score. Secondary outcomes are mobility measured by the Elderly Mobility Scale, functional performance, quality of life, return to home rate, level of pain, type and dosage of analgesic medications, the number of falls after treatment, the number of (fracture-related) complications, 1-year and 2-year mortality. Every 6 weeks, a cluster will switch from current practice to the clinical pathway. The aim is a total of 393 inclusions, which provides an 80% statistical power for an improvement in mobility of 10%, measured by the Parker mobility score.

**Ethics and dissemination:**

The Medical Research Ethics Committee of Academic Medical Center has exempted the PELVIC study from the Medical Research Involving Human Subjects Act (WMO). Informed consent will be obtained using the opt-out method and research data will be stored in a database and handled confidentially. The final study report will be shared via publication without restrictions from funding parties and regardless of the outcome.

**Trial registration number:**

NCT06054165.

**Protocol version:**

V.1.0, 19 July 2022

**STRENGTHS AND LIMITATIONS OF THIS STUDY**

- The stepped-wedge design allows for the systematic implementation of multiple interventions in various aspects of the diagnosis and treatment strategy for pelvic fragility fractures, providing a holistic approach to addressing this multifactorial problem.
- Focusing on mobility as the primary outcome, rather than mortality, aligns with the priorities of older adult patients and demonstrates a patient-centred approach.
- The stepped-wedge design facilitates more targeted and coordinated visits at the participating centres, improving adherence to the protocol compared with a regular randomised controlled trial design.
- The stepped-wedge design introduces complexity into the statistical analysis due to the sequential introduction of clusters, making data analysis more challenging.
- The multifaceted approach in the stepped-wedge design makes it difficult to determine which specific intervention has the most significant impact on outcomes, hindering the identification of the most effective strategies.

## Introduction

 The Dutch population is rapidly ageing. In January 2024, 20.5% of the Dutch population was 65 years and older, compared with 12.8% in 1990.^1^ This increase in older adults was also reflected in the incidence of pelvic fragility fractures (FFPs), which saw an age-adjusted increase of 399% between 1970 and 2013.^2^ This trend is expected to persist with the ageing population.

FFPs, also known as osteoporotic pelvic fractures, pose a serious burden on our healthcare system, with a 1-year mortality rate of 10%–27%, mainly caused by complications related to immobility.^3^ The burden on patients with an FFP extends beyond mortality and complications; many experience a significant reduction in mobility, loss of independence in activities of daily living (ADL) and high pain levels. Consequently, the total number of hospital admissions saw an increase of 127% between 1986 and 2011,^4^ and only 25%–56% of these patients are able to return to their own home after hospitalisation.^5^

Despite the rising incidence, evidence on the optimal diagnostic and treatment strategies is lacking. Additionally, there is currently no evidence-based treatment guideline implemented in the Netherlands, leading to a wide variety of clinical practices.^6^ This variation is apparent in diagnostic strategies; for example, the routine use of CT to detect FFP is not common practice in many hospitals in the Netherlands, even though concomitant posterior pelvic ring lesions are missed in 32%–97% of the patients with pubic rami fractures on radiographs.^7^,^9^ Accurately identifying the full scope of the fracture pattern has gained clinical importance due to the development of minimally invasive percutaneous techniques, which have lowered the threshold for surgery in older adult patients.

Although surgical techniques have improved over the last few years, determining who and when to operate on remains an international subject of debate. The Rommens classification is intended to reflect the increasing degree of instability and the need for surgical fixation.^10^ However, recent systematic reviews show that selecting patients for surgery based solely on radiographic criteria does not lead to the best outcomes and advise to take pain and loss of mobility into consideration as criteria for surgical fixation.^311^,^14^ In recent literature, several clinical pathways have been proposed to guide diagnostic and treatment strategies.^15^,^20^ Some of these pathways rely on radiographic classifications like the Rommens classification^10^ while others assess clinical function by evaluating the patient’s mobility. However, none of these pathways have significantly improved patient outcomes and all seem to target a singular underlying problem in the diagnosis and management of these patients. One study shows promising results when surgically fixating patients with FFP type 1 or type 2 if they suffer immobilising or prolonged pain without adequate mobilisation after 5–7 days, and FFP types 3 and 4.^21^ Mortality was low (<10%), and 85% were able to return to their own home.^21^

Furthermore, many clinicians still believe that surgical intervention is too invasive for these patients or will not improve their outcomes so patients are rarely referred to specialised hospitals for surgical fixation.^6^ Additionally, the follow-up period is often short or non-existent for non-operatively treated patients, overlooking the problem of fracture progression, which is observed in 14% of all FFP patients.^7 22^

To summarise, these patients face multifactorial problems in their diagnostic and treatment strategies, and improving only one aspect will only partially improve patient outcomes. This underscores the importance of a holistic approach to address the multifactorial nature of this problem.

The PELVIC study is a closed cohort stepped wedge cluster randomised design, which involves a sequential crossover of clusters with the order of crossover randomly determined. This study will be conducted with a superiority design and aims for regional implementation of an evidence-based and expert opinion-based clinical pathway. The primary objective of this study is to evaluate whether the implementation of a clinical pathway can improve short-term mobility (<6 weeks) in all patients ≥50 years who sustained an FFP after low-energy trauma (LET). The secondary objectives are to assess the effect on functional performance, quality of life, return to home rate, level of pain, type and dosage of analgesic medications used, falls after hospital discharge, (fracture-related) complications, 1-year and 2-year mortality rate including (presumed) cause of death.

## Methods and analysis

The Standard Protocol Items: Recommendations for Interventional Trials checklist was used when writing this report.^23^

### Study setting

This study will be a regional stepped-wedge cluster randomised controlled trial (RCT) which aims to implement a clinical pathway in nine trauma centres (academic and non-academic, levels 1, 2 and 3) that are part of the trauma networks of Netwerk Acute Zorg Noord-Holland/Flevoland in the Netherlands. Within this referral network, the Amsterdam UMC and Noordwest Ziekenhuis are already functioning as tertiary referral hospitals regarding pelvic and acetabular surgery. The trial will be designed in adherence to the Consolidated Standards of Reporting Trials statement for cluster randomised trials and extension for stepped-wedge trials.^24 25^ During the design of the PELVIC study, a focus group was set up to make sure relevant specialties involved in this clinical pathway were represented and all aspects of care were as optimal as possible. The focus group included among others several specialised pelvic surgeons, musculoskeletal radiologists, clinical geriatrician and a physiotherapist specialised in trauma rehabilitation. Multiple separate and one conjoined meetings were held to achieve consensus on the final design of the clinical pathway.

In a stepwise manner, each cluster will cross over from control (current practice) to intervention (clinical pathway) phase. Each cluster contains one trauma centre so the number of sequences is equal to the number of participating centres. Nine trauma centres will participate in the study: Amsterdam UMC, BovenIJ Ziekenhuis, Dijklander Ziekenhuis, Flevoziekenhuis, NoordWest Ziekenhuis, OLVG, Spaarne Gasthuis, Zaand Medisch Centrum, Ziekenhuis Amstelland.

### Patient and public involvement

No patients or public were involved in the design, or conduct, or reporting, or dissemination plans of our research. However, we do monitor patient satisfaction about the clinical pathway during the follow-up visits and will publish these results regardless of the outcome.

### Eligibility criteria

All older adult patients (≥50 years old) who suffered an FFP after LET and are presented to the emergency room or outpatient clinic of one of the participating hospitals are eligible for inclusion. Pelvic fractures after LET only occur in/most often relate to patients with osteoporosis and can thus be classified as fragility fracture. To ensure the study represents the day-to-day reality of treating these patients as closely as possible, and to make the results the most relevant for clinical practices, the investigators decided to include patients who were presented at the emergency room of one of the participating centres as well as patients who had a delay in presentation due to prior treatment elsewhere. Patients of both sexes and all ethnicities with an FFP can participate, as long as patients understand the physiotherapy instructions for early mobilisation. Therefore, patients who have severe cognitive decline or insufficient comprehension of the Dutch language are excluded. Patients with high suspicion of a pelvic fracture caused by a malignant tumour, or who receive palliative or terminal care, are wheelchair users or bedridden are also excluded from the study. Patients who present to the emergency room, because they suffer from complications from previous pelvic ring fixation, are also excluded.

### Interventions

**Figure 1:**
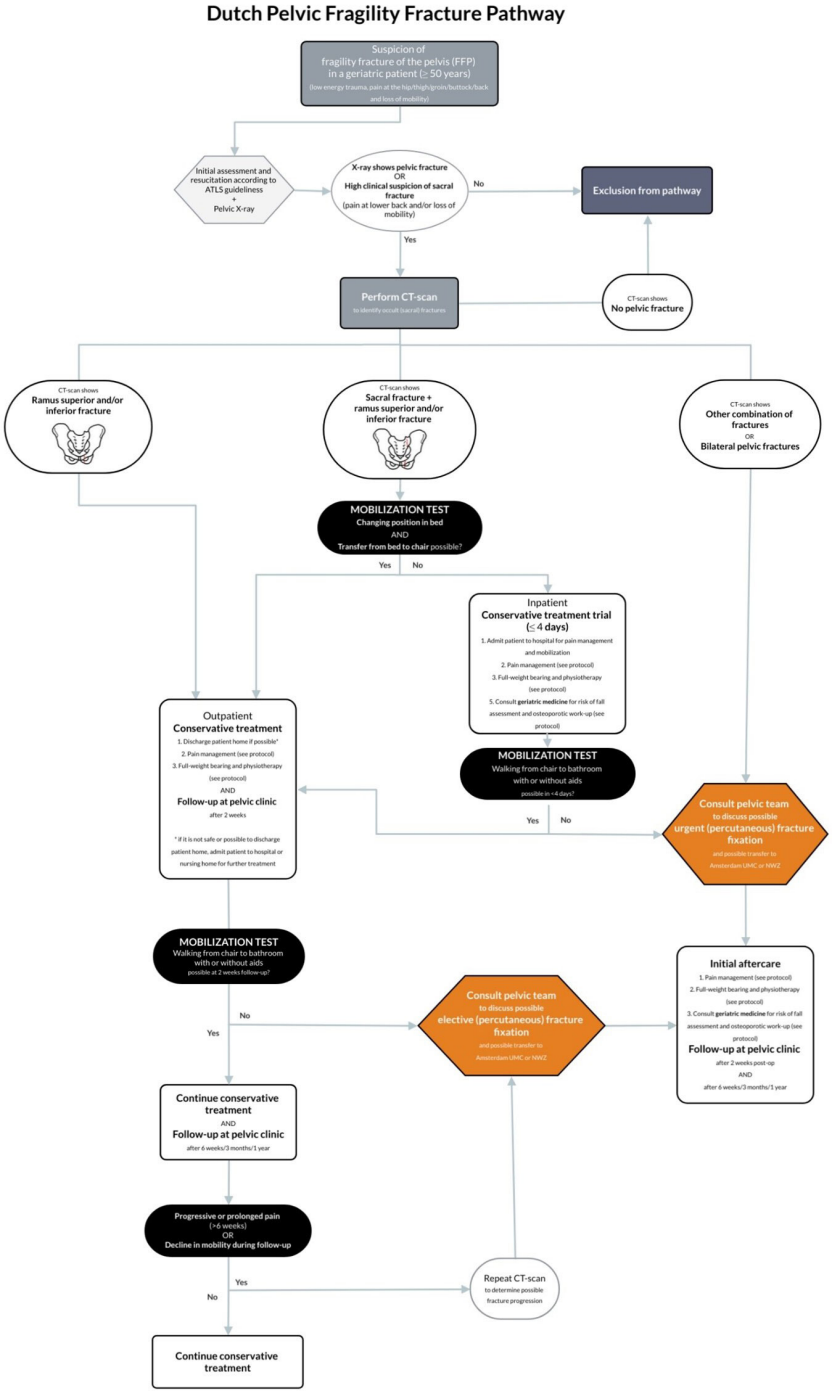
Details of clinical pathway of PELVIC study.

To determine the optimal clinical pathway, the investigators identified previously proposed clinical pathways in recent literature and expert opinions from the FFP focus group. The pathway could not be based on previous guidelines since there is currently no evidence-based guideline on FFP in the Netherlands. This resulted in the following clinical pathway (see figure 1). The pathway aims to optimise the diagnostic process, guides the decision-making process for further treatment (eg, operative or conservative), structures the follow-up process so fracture progression will not be missed and can be treated and aims for a lower threshold for referrals to pelvic expertise centres if deemed necessary. Furthermore, guidelines on postoperative care, pain management, a physiotherapy protocol to promote early mobilisation, and a geriatric and osteoporosis workup will be provided. All the advice that is given is based on the care that these patients in some centres already receive, no new and experimental product or treatment is introduced. There will be no restriction on the use of (co)medication or other kind of interventions, with the exception of pain medication prescribed as part of the management of the pelvic fracture.

Because of the stepped-wedge cluster RCT design, it is important that all participating randomised trauma centres complete the trial. If they do not complete the trial, this will lead to an unequal distribution of patients between current practice and clinical pathway arms. If a centre does drop out of the study, the randomisation order will be maintained. Patients treated in a centre that dropped out during this trial will still be accounted for in the final analysis, according to intention-to-treat analysis. Individual subjects can leave the study at any time for any reason if they wish to do so without any consequences. The investigator can decide to withdraw a subject from the study for urgent medical reasons. Individual subjects who decide to withdraw from the PELVIC study will not be replaced by new subjects.

The choice of a stepped-wedge design was based on the importance of a correct implementation and optimal adherence to the design of the clinical pathway in the participating centres. In a ‘normal’ RCT, multiple clusters will cross over from control to intervention phase at the same time, which makes it very hard to effectively inform all centres about the clinical pathway in time. A stepped-wedge structure allows for more targeted visits during the wash-in period to ensure all involved are educated about the pathway and its design in time. Informing a centre too early in the study will contaminate the results of the control phase, which is undesirable.

### Outcome measurements and data collection

The primary outcome is mobility measured by the Parker Mobility Score (PMS).^26^ Since there is no validated tool to measure mobility specifically after a pelvic fracture, the investigators choose to use The PMS as a primary outcome measurement. The PMS is a valid and reliable score measuring mobility in hip fracture patients. The PMS answers three questions, each valued 0–3 points and is commonly used in clinical practices to monitor the mobility of geriatric patients. A score of 0–3 is considered low, 4–6 moderate and 7–9 reflects good mobility. Furthermore, the PMS is a validated assessment tool for mortality in patients with reduced mobility after hip surgery. The PMS will be measured a change from baseline at 2 weeks, 3 months, 6 months and 1 year. The preinjury PMS will be retrospectively assessed at baseline or after 2 weeks.

The following secondary outcome measures will be used:

1. Mobility, using the modified Elderly mobility scale (EMS) at 2 weeks, 3 months and 1 year.^27^ The preinjury EMS will be retrospectively assessed at baseline or after 2 weeks. The EMS is a 20-point validated assessment tool to evaluate mobility specifically in older adult patients. The investigators decided, despite the fact that this tool has an element of measurement in it which makes it harder to determine the preinjury score, to add this tool since it does a better job distinguishing between patients who are able to sit up and go from sitting to standing. The EMS ranges from 0 to 20 points, with high scores representing better outcomes than low scores.
2. Functional performance, using the Katz Index of Independence in ADL (KATZ ADL) after 3 months and 1 year.^28^ The preinjury KATZ ADL will be retrospectively assessed after 3 months. This index is one of the most commonly used scores to measure the functional status of older adult individuals. It assesses the ADL using six questions, each valued 0 or 1. The score ranges from 0 to 6, and a score of 6 indicates full function while 4 indicates moderate impairment and 2 or less indicates severe functional impairment
3. Quality of life, using the EuroQol-5 Dimension-5 Level (EQ-5D-5L) at 1 year.^29^ The EQ-5D-5L is a generic quality of life questionnaire which consists of a Visual Analogue Scale and five questions about mobility, self-care, usual activities, pain or discomfort and anxiety or depression. Each question has three answer alternatives, with 1 indicating the optimal health state and 5 indicating severe problems. There are 3125 possible health states defined by combining one level from each dimension, ranging from 11 111 (full health) to 55 555 (worst health).
4. Return to home rate at baseline (discharge), after 6 weeks, 3 months, 6 months and 1 year. The residencies will be grouped into ‘living independently at home’, ‘assisted living’, ‘nursing home’, ‘rehabilitation centre’ and ‘palliative care facility’.
5. Level of pain, using the Numerical Pain Rating Scale (NRS) at baseline (hospital admission), 1-day postoperative (if the patient underwent surgery), at 2 weeks, 6 weeks, 3 months and 1 year.^30^ The Numerical Pain Rating Scale is a specific measurement tool from 0 to 10, with 0 reflecting no pain, 1–4 mild pain, 5–7 moderate pain and 7–10 severe pain. This tool is currently already used by nurses in all hospitals in the Netherlands.
6. Descriptive name and dosage of analgesic medications used at baseline (discharge), at 2 weeks, 6 weeks, 3 months and 1 year. The preinjury dosage and type of analgesic medication will be retrospectively assessed at baseline or after 2 weeks. All analgesic medication that the patient is given will be recorded, and compared with the analgesic medication that the patient used prior to injury. The medication will be categorised according to the WHO analgesic ladder to facilitate comparison of changes in patients’ analgesic medication usage.^31^
7. Falls after hospital discharge at 6 weeks and 3 months. The investigators will ask the patients if they fell since hospital discharge, how many times and if any of these falls resulted in additional injury in need of medical attention (including hospitalisation). Falls and the frequency of falling are related to an increased risk of mortality and subsequent fracture risk in older adult patients.^32^
8. The number of participants with complications (fracture related) at 2 weeks, 3 months, 6 months and 1 year. This includes general complications that may occur during a period of reduced mobility or hospital admission (such as pneumonia, urinary tract infection, thromboembolic event, heart failure, cerebrovascular event and myocardial infarction). Complications related to operative treatment will also be recorded. These complications include but are not limited to reoperation, bleeding, delayed operation, infection, screw back out, malposition of screw and neurological damage. All complications will be categorised according to level of severity and the necessity for further treatment according to the Clavien-Dindo classification.^33^
9. One-year mortality including (presumed) cause of death.
10. Two-year mortality including (presumed) cause of death.

Other study parameters to be collected are as follows:

Patient characteristics:

- Age at trauma in years (calculated from date of birth and date of trauma).
- Sex (male or female).
- Body mass index (calculated using length and weight).
- American Society of Anesthesiologists (ASA) grade (I, II, III, IV, V or unknown).
- Comorbidities (using the Charlson Comorbidity Index^34^).
- Medication use prior to trauma.
- Osteoporosis treatment prior to trauma.
- Previous type of residence (independent living/assisted living/nursing home/care facility).

Injury characteristics:

- Mechanism of injury (descriptive).
- Affected side (left/right/bilateral).
- Fracture pattern, classified using the Young and Burgess classification,^35^ Rommens classification^10^ and OF-Pelvis classification.^36^
- Additional injuries (descriptive).

Treatment characteristics:

- Date of first presentation at emergency room or outpatient clinic.
- Treatment received for pelvic fracture prior to presentation (descriptive).
- Length of hospital admission (calculated from date of admission and discharge).
- Discharge location (independent living/assisted living/nursing home/care facility/palliative care facility).
- Hospital of admission (descriptive).
- Date of admission to hospital that patient is transferred to for surgical fixation.
- Time to surgery (calculated from dates of trauma and date of surgery).
- Details of surgery (minimally invasive/open/plate/screw).
- Indication for surgery (fracture pattern, pain, mobility, comorbidities).
- Operative time (in minutes).
- Type of anaesthesia (general anaesthesia or regional epidural/spinal anaesthesia).
- Peripheral nerve block (yes/no).
- Details of non-operative treatment (descriptive).
- Adherence to early mobilisation orders (yes/no/unclear).
- Physiotherapy consulted inpatient (yes/no).
- Physiotherapy consulted outpatient (yes/no).
- Geriatric medicine specialist consulted (yes/no).
- Pain medication administered conforms to advised pain protocol (yes/no).

Pelvic CT imaging

- Presence of bone oedema on dual energy imaging (abnormal increase in HU signal intensity).
- Distribution of bone oedema on dual energy imaging (bone site).
- Fracture progression on follow-up imaging measured as increased dislocation of fracture pattern (difference in fracture dislocation in millimetres on initial imaging and follow-up imaging).
- Additional pelvic fractures on follow-up imaging (yes/no, location).
- Bone union (normal/delayed/non-union/malunion).
- Plain radiograph imaging done during follow-up (yes/no).
- CT imaging done during follow-up (yes/no).
- Complications of osteosynthese material on follow-up imaging (screw backing out, screw breakage, loosening of osteosynthese material, other).

### Study timeline

At the start of the study, all clusters will be in the control phase for 6 weeks. After 60 weeks, all clusters will have crossed over to the intervention phase and will remain in the intervention phase for another 6 weeks. The total duration of the trial will be 66 weeks and is determined by the number of participating centres and the required sample size. Details of the sample size calculation are described in the ‘Sample size calculation’ section. The order in which the clusters will cross over is randomised. To achieve effective implementation of the clinical pathway, a structured 2-week wash-in phase was designed. In this time frame, the study team will discuss with the local hospital how to implement the clinical pathway efficiently. It is important to avoid contamination of the data for clusters still in the control phase. Details on the clinical pathway will, therefore, not be shared with local clinicians before the transfer to the intervention phase. In the analysis of this study, every cluster is their own control because of the cluster RCT design.

### Sample size

The aim is a total of 393 inclusions, which provides an 80% statistical power for an improvement in mobility of 10%, measured by the PMS. An improvement in mobility is defined as an increase in the number of patients with a preinjury PMS of 9–6 (‘high mobility group’) who have a minimum of PMS 6 after treatment, and the patients with a preinjury PMS of 5 or lower (‘low mobility group’) who regain their old PMS. This requires a sample size of 197 per group or 393 patients in total.

### Recruitment

The aim of the PELVIC study is to implement a clinical pathway by educating and stimulating local clinicians. Besides the targeted visits in the wash-in phase, no specific efforts will be made to achieve adequate participant enrolment.

### Allocation and blinding

Randomisation and allocation of the participating centres will be performed by the lead researcher using R statistics software. The randomisation sequence is unknown to all participating centres and clinicians, and centres were informed in a timely manner about the moment to switch to intervention by the lead researcher of this study. Although there is allocation concealment, this study is otherwise an open-label trial since subjects and physicians are aware of the assigned treatment (current practice vs clinical pathway).

### Data management and monitoring

Any information collected during this study will be encoded and stored in a password-protected database with restricted access to the researcher team only. Data will be entered once. The quality of the entered data will be monitored by checking entry for a random sample of patients prior to database locking. There will be no data safety monitoring board. This study does not introduce an investigational product or experimental intervention so no additional safety reporting will be performed.

### Statistical methods

Outcomes of all patients with an FFP will be analysed before and after the implementation of the clinical pathway. Patients will be assigned to current practice or clinical pathway cohort based on the date of first hospital contact (eg, emergency room visit, outpatient clinic visit, hospital admission date). When patients are diagnosed in a ‘clinical pathway’ centre but transferred to a pelvic expertise centre for fixation that is still in current practice, the patient will be assigned to the clinical pathway cohort. Data will be analysed by using the SPSS V.24.0 or higher (SPSS. Normality of continuous data will be tested with the Shapiro-Wilk test. Homogeneity of variances will be tested using the Levene’s test. A two-sided p<0.05 will be taken as threshold of statistical significance in all statistical tests. Primary analysis will be performed with an intention-to-treat analysis according to the randomisation order and cross-over dates. If implementation is not performed as scheduled, secondary analysis will be performed according to a per-protocol analysis. The primary comparison between current practice and clinical pathway will be performed for patients from all hospitals participating in the PELVIC study. A secondary sensitivity analysis will be done to compare the outcomes when excluding the hospitals that are currently operating and conform a protocol very similar to the clinical pathway. Another sensitivity analysis will be done where the outcomes of the patients with an isolated ramus fracture will be excluded since the advice for treatment in the clinical pathway of this subgroup is very similar to the current practice. Missing data on baseline characteristics will be imputed by multiple imputation techniques. Outcome data will not be imputed, patients who died or are lost to follow-up within 1 year will remain in the analysis as censored at the date of loss to follow-up. Complete and multiple imputed data analyses will be performed to check for inconsistencies.

## Ethics and dissemination

The Medical Research Ethics Committee (MREC) of Academic Medical Center confirmed that the Medical Research Involving Human Subjects Act (WMO) does not apply to the above-mentioned study and that an official approval of the study is not required. Important protocol modifications will be notified to the MREC of the participating centres.

Informed consent will be obtained using the opt-out method to minimise the significant information and selection bias that will otherwise occur.

Research data will be stored in a database (eg, Castor Electronic Data Capture or a similar platform that meets Good Clinical Practice standards) and will be handled confidentially. Any information collected during this study on paper will be placed in a research folder, which will be filed in locked cabinets in research offices at the participating hospitals. Any electronic information will be saved in a password-protected area; only the study staff will have access to these data. Research data that can be traced to individual persons can only be viewed by authorised personnel.

Prior to the start of the study, the PELVIC study was registered in an online database of clinical research studies (ClinicalTrials.gov). The final study report will be shared via Open Access publication without restrictions from funding parties and regardless of the outcome.
