# Supplementary material for: Does regional implementation of a clinical pathway for older adult patients with pelvic fragility fractures after low-energy trauma improve patient outcomes (PELVIC): a multicentre, stepped-wedge, randomised controlled trial
Source: BMJ Open. 2024 Aug 13;14(8):e083809. doi: 10.1136/bmjopen-2023-083809 (PMC11331913; doi:10.1136/bmjopen-2023-083809)
Supplement: Supplementary file 1 [file bmjopen-2023-083809.reviewer_comments.pdf]

PEER REVIEW HISTORY

BMJ Open publishes all reviews undertaken for accepted manuscripts. Reviewers are asked to complete a checklist review form and are provided with free text boxes to elaborate on their assessment. These free text comments are reproduced below.

ARTICLE DETAILS

**TITLE (PROVISIONAL)**	Does regional implementation of a clinical pathway for older adult patients with pelvic fragility fractures (PELVIC) after low energy trauma improve patient outcomes; a multicentre, stepped-wedge, randomized controlled trial
**AUTHORS**	Mennen, Anna; Lommerse, Marte; Hemke, Robert; Willems, Hanna; Maas, Mario; Bloemers, Frank; Ponsen, Kees Jan; Van Embden, Daphne

VERSION 1 - REVIEW

**REVIEWER NAME**	Cedeño Veloz, Bernardo Abel
**REVIEWER AFFILIATION**	Navarrabiomed, Geriatric Department
**REVIEWER CONFLICT OF INTEREST**	None.
**DATE REVIEW RETURNED**	20-Jan-2024

**GENERAL COMMENTS**	Despite being a well-conceived protocol, there are significant flaws in the current manuscript<br><br>1 Avoid use of elderly. It is older adults<br>2 Although I am not sure that individuals ≥ 50 years old can be considered older adults.<br>3 Miss important parts in a protocol manuscript:<br>- No discussion section<br>- Contribution to the Field<br>- Trial Status<br>- Schedule table<br>4 Few and outdates references<br><br>Minor comments:<br>The introduction is informative and effectively establishes the importance and relevance of the study. It could be made more concise, especially in the parts discussing the burden and complications of pelvic fractures. Providing context or comparative data regarding clinical practices in other countries could enhance the argument about the variability in clinical practice and the need for standardized guidelines.

**REVIEWER NAME**	Rommens, Pol Maria
**REVIEWER AFFILIATION**	University Medical Center of the Johannes Gutenberg University Mainz
**REVIEWER CONFLICT OF INTEREST**	No competing interest
**DATE REVIEW RETURNED**	23-Jan-2024

GENERAL COMMENTS	Page 6/30, line 30. Please insert „Nanninga GL, de Leur K, Panneman MJ, van der Elst M, Hartholt KA. Increasing rates of pelvic fractures among older adults: The Netherlands, 1986-2011. Age Ageing. 2014 Sep;43(5):648-53. doi: 10.1093/ageing/aft212. Epub 2014 Jan 12. PMID: 24419459.“ as reference.<br>Page 7/30, line 21: Please insert reference 18 “Rommens PM, Hofmann A. Comprehensive classification of fragility fractures of the pelvic ring: Recommendations for surgical treatment. Injury. 2013 Dec;44(12):1733-44. doi: 10.1016/j.injury.2013.06.023. Epub 2013 Jul 18. PMID: 23871193“ here.<br>Page 7/30, line 38 and line 52: Please insert reference “Rommens PM, Arand C, Hopf JC, Mehling I, Dietz SO, Wagner D. Progress of instability in fragility fractures of the pelvis: An observational study. Injury. 2019 Nov;50(11):1966-1973. doi: 10.1016/j.injury.2019.08.038. Epub 2019 Aug 27. PMID: 31492514” here.<br>Page 7/30, line 51: to significantly improve (instead of improvement) patient outcomes<br>Page 8/30, line 7: please insert “Oberkircher L, Ruchholtz S, Rommens PM, Hofmann A, Bücking B, Krüger A. Osteoporotic Pelvic Fractures. Dtsch Arztebl Int. 2018 Feb 2;115(5):70-80. doi: 10.3238/arztebl.2018.0070. PMID: 29439771; PMCID: PMC5817189“ here.<br>Page 8/30, line 8: please insert reference 18 here<br>Page 8/30, line 13: I agree that there is confusing data. Nevertheless, some papers suggest a better outcome after operative treatment. In the paper of Wagner et al, a mortality below 10% and very high rate of patients returning home is found in patients treated with sacral bar.<br>Page 9/30, line 55: You take a threshold of 50 years for inclusion of patients with FFP. Why do you take this low threshold? Elderly or geriatric patients are generally regarded as patients of 65 years of age or above. Most patients of age 50 and above still have a robust skeleton with good bone mineral density. It is unlikely that these patients will suffer a FFP due to low energy trauma (LET) of even unknown trauma. Please justify this choice.<br>Page 10/30, line 34: please insert references 7-11 and “Oberkircher L, Ruchholtz S, Rommens PM, Hofmann A, Bücking B, Krüger A. Osteoporotic Pelvic Fractures. Dtsch Arztebl Int. 2018 Feb 2;115(5):70-80. doi: 10.3238/arztebl.2018.0070. PMID: 29439771; PMCID: PMC5817189“ here.<br>Page 10/30, line 39: In figure 1. Will the participating centres not be allowed to treat these patients operatively. Are they obliged to transfer the patients for operative treatment to the 2 “pelvic” centres?<br>Page 10/30, line 39: In figure 1, you seem to follow the FFP classification (see reference 18) – you use the figures from this publication - for your algorithm. This brings up two questions: 1. How do you recommend to treat patients with posterior lesions without anterior fracture (FFP type IIa)? 2. Do you make a difference between nondisplaced (type IIb and IIc) and displaced unilateral fractures (FFP type IIIb and c)?<br>Page 12/30, line 32. Please add a reference<br>Page 12/30, line 49: please add reference for Katz Index<br>Page 13/30, line 33: please add reference for NRS<br>Page 13/30, line 47: there is a large spectrum of medications available on the market, which makes comparison of type and dosage very difficult. It is suggested to categorize the medication into the three levels, given by the World Health Organization (WHO).

	This makes it easier to see if the patient has gone from a level of stronger medication to a level of milder medication.<br>Page 14/30, line 18: replace tot by that<br>Page 15/30, lines 20-25: using 3 classification systems makes it much more complex for the study centres and the final analysis<br>Page 15/30, line 51: recent biomechanical studies sow that there is a large difference in stability provided by unilateral sacroiliac screws, cement-augmented unilateral sacroiliac screws and transsacral screws or bars. Which treatment technique do you recommend or follow? In case different techniques are used, it will be difficult to analyse which technique provides the best outcome for the patient, due to low patient numbers in each category.<br>Page 16/30, line 58: 60 weeks???

VERSION 1 – AUTHOR RESPONSE

Reviewer: 1

Dr. Bernardo Abel Cedeño Veloz, Navarrabiomed

Comments to the Author:

Despite being a well-conceived protocol, there are significant flaws in the current manuscript

1 Avoid use of elderly. It is older adults

Thank you for your feedback, we have changed the term elderly towards older adult as this carries less of a connotation of frailty and dependency, which can be seen as derogatory.

2 Although I am not sure that individuals ≥ 50 years old can be considered older adults.

Defining who qualifies as an “older adult” remains topic of debate. In the context of osteoporosis guidelines and major hip fractures studies, the threshold is often set at 50 years and older. This inclusion criterion underscores the importance of considering age-related changes in bone mineral density and fracture risk starting at midlife.

3 Miss important parts in a protocol manuscript:

- No discussion section

- Contribution to the Field

- Trial Status

- Schedule table

Thank you for your feedback. According to the BMJ Open submission guidelines, the sections "Discussion," "Contribution to the Field," "Trial Status," and "Schedule Table" are not explicitly required for study protocols. Therefore, we have chosen not to include them in our manuscript at this time. However, if the absence of these sections impacts the eligibility of our paper for publication, we

are willing to reconsider and incorporate the requested information as needed. Please let us know how you would like to proceed. Thank you for your understanding.

4 Few and outdates references

We have updated the references with the suggestions from Reviewer 2. Please indicate if any relevant references are still missing, we are more than willing to add them to the manuscript.

Minor comments:

The introduction is informative and effectively establishes the importance and relevance of the study. It could be made more concise, especially in the parts discussing the burden and complications of pelvic fractures. Providing context or comparative data regarding clinical practices in other countries could enhance the argument about the variability in clinical practice and the need for standardized guidelines.

We appreciate your feedback on the introduction and have rewritten it to make it more concise and relevant, and have referenced the existing clinical pathways to highlight the clinical practice variability and explained how they differ from the pathway we propose.

Reviewer: 2

Dr. Pol Maria Rommens, University Medical Center of the Johannes Gutenberg University Mainz

Comments to the Author:

Page 6/30, line 30. Please insert „Nanninga GL, de Leur K, Panneman MJ, van der Elst M, Hartholt KA. Increasing rates of pelvic fractures among older adults: The Netherlands, 1986-2011. Age Ageing. 2014 Sep;43(5):648-53. doi: 10.1093/ageing/aft212. Epub 2014 Jan 12. PMID: 24419459." as reference.

Thank you for your comment. Due to different formatting line 30 on page 6 does not correspond to information related to the article of Nanninga et al. However, as we agree this is a relevant article in our area of expertise, opted to include the reference and data about the reference in the introduction.

"Consequently, the total number of hospital admissions saw an increase of 127% between 1986 and 2011,"

Page 7/30, line 21: Please insert reference 18 "Rommens PM, Hofmann A. Comprehensive classification of fragility fractures of the pelvic ring: Recommendations for surgical treatment. Injury. 2013 Dec;44(12):1733-44. doi: 10.1016/j.injury.2013.06.023. Epub 2013 Jul 18. PMID: 23871193" here.

We have added the relevant reference to our manuscript.

Page 7/30, line 38 and line 52: Please insert reference "Rommens PM, Arand C, Hopf JC, Mehling I, Dietz SO, Wagner D. Progress of instability in fragility fractures of the pelvis: An observational study. Injury. 2019 Nov;50(11):1966-1973. doi: 10.1016/j.injury.2019.08.038. Epub 2019 Aug 27. PMID: 31492514" here.

We have added the relevant reference to our manuscript.

Page 7/30, line 51: to significantly improve (instead of improvement) patient outcomes

Thank you very much for pointing out this oversight. The mistake has been corrected when we rewrote the introduction.

Page 8/30, line 7: please insert “Oberkircher L, Ruchholtz S, Rommens PM, Hofmann A, Bücking B, Krüger A. Osteoporotic Pelvic Fractures. Dtsch Arztebl Int. 2018 Feb 2;115(5):70-80. doi: 10.3238/arztebl.2018.0070. PMID: 29439771; PMCID: PMC5817189“ here.

We have added the relevant reference to our manuscript.

Page 8/30, line 8: please insert reference 18 here

We have added the relevant reference.

Page 8/30, line 13: I agree that there is confusing data. Nevertheless, some papers suggest a better outcome after operative treatment. In the paper of Wagner et al, a mortality below 10% and very high rate of patients returning home is found in patients treated with sacral bar.

Thank you for your comment. We have added the relevant reference to our manuscript and described the data.

Page 9/30, line 55: You take a threshold of 50 years for inclusion of patients with FFP. Why do you take this low threshold? Elderly or geriatric patients are generally regarded as patients of 65 years of age or above. Most patients of age 50 and above still have a robust skeleton with good bone mineral density. It is unlikely that these patients will suffer a FFP due to low energy trauma (LET) of even unknown trauma. Please justify this choice.

Defining the threshold age for inclusion in studies about osteoporosis is indeed topic of debate. In the context of osteoporosis guidelines and major hip fractures studies, the threshold is often set at 50 years and older. This inclusion criterion underscores the importance of considering age-related changes in bone mineral density and fracture risk starting at midlife. We agree that the number of patients who suffer from a FFP due to LET at the age of 50 is very low, but it is not impossible. To avoid picking an arbitrary age, we decided to determine the age of inclusion on these criteria.

Page 10/30, line 34: please insert references 7-11 and “Oberkircher L, Ruchholtz S, Rommens PM, Hofmann A, Bücking B, Krüger A. Osteoporotic Pelvic Fractures. Dtsch Arztebl Int. 2018 Feb 2;115(5):70-80. doi: 10.3238/arztebl.2018.0070. PMID: 29439771; PMCID: PMC5817189“ here.

Due to different formatting line 34 on page 10 does not correspond to information related to this article. If possible, could you please specify where this reference is missing so I can add it in the paper.

Page 10/30, line 39: In figure 1. Will the participating centres not be allowed to treat these patients operatively. Are they obliged to transfer the patients for operative treatment to the 2 “pelvic” centres?

Thank you for your comment. In the Netherlands pelvic fracture surgery is very much centralised care and is only carried out in mostly level 1 hospitals due to Dutch government guidelines that state that pelvic and acetabular fracture surgery can only be performed if hospitals meet specific surgical volume and facilitate specific criteria (e.g. interventional radiology). In our region, only 2 hospitals are allowed to carry out these surgical procedures. So if surgical fixation is necessary, patients are transferred to a 'pelvic surgery centre'. These referral networks are in line with our trauma regions and have been in place for many years and is not part of the study. To add, since the Netherlands is such a small country this is not a major logistic problem. We did notice from our previous survey study that surgeons are not likely to refer patients for surgical fixation at the moment for many reasons, mainly because they deem surgical fixation to invasive [1]. Discussing cases with a pelvic expert team is therefor part of the intervention in this study, so the indication for surgical fixation can be discussed with the team that carries out the procedure and patients are not unnecessarily deemed unfit for surgery.

Page 10/30, line 39: In figure 1, you seem to follow the FFP classification (see reference 18) – you use the figures from this publication - for your algorithm. This brings up two questions: 1. How do you recommend to treat patients with posterior lesions without anterior fracture (FFP type IIa)? 2. Do you make a difference between nondisplaced (type IIb and IIc) and displaced unilateral fractures (FFP type IIIb and c)?

Thank you for your questions regarding our treatment algorithm.

Treatment of patients with posterior lesions without anterior fracture (FFP Type IIa): For FFP Type IIa, we allow a few days (3-5) for patients to optimize their mobility with weightbearing as tolerated. If there is no significant improvement in mobility within this period, surgical fixation is recommended.

Distinction between nondisplaced (Type IIb and IIc) and displaced unilateral fractures (FFP Type IIIb and IIIc): Both displaced and non-displaced fractures (Type IIb&IIc and IIIb&IIIc) are treated monitored closely and are allowed a few days of weightbearing as tolerated to optimize mobility. If patients fail to regain mobility within 3-5 days, surgical fixation is recommended.

Summary of our approach:

- Isolated Anterior Fractures: Generally non-operative, surgery in case of fracture progression
- Bilateral Fractures: Immediate expert consultation.
- Other Fractures: Surgery considered if no improvement in mobility within five days.

Page 12/30, line 32. Please add a reference

We have added the appropriate reference for this information.

Page 12/30, line 49: please add reference for Katz Index

We have added the appropriate reference for this information.

Page 13/30, line 33: please add reference for NRS

We have added the appropriate reference for this information.

Page 13/30, line 47: there is a large spectrum of medications available on the market, which makes comparison of type and dosage very difficult. It is suggested to categorize the medication into the three levels, given by the World Health Organization (WHO). This makes it easier to see if the patient has gone from a level of stronger medication to a level of milder medication.

Thank you for your insightful suggestion. We will follow your recommendation to categorize the medication according to the WHO pain ladder and have added this to the protocol.

Page 14/30, line 18: replace tot by that

Thank you for your remark, we have changed the spelling of tot to that.

Page 15/30, lines 20-25: using 3 classification systems makes it much more complex for the study centres and the final analysis

We agree that using multiple classification systems is more complex than using 1. However, for the clinical pathway which is used by the participating centres only 1 is used for simplicity. For the data collection however multiple will be used to gain more insight in how the different classifications reflect in this patient population and their outcomes. This data collection will be done by a dedicated pelvic fracture researcher, so complexity is less of an issue.

Page 15/30, line 51: recent biomechanical studies sow that there is a large difference in stability provided by unilateral sacroiliac screws, cement-augmented unilateral sacroiliac screws and transsacral screws or bars. Which treatment technique do you recommend or follow? In case different techniques are used, it will be difficult to analyse which technique provides the best outcome for the patient, due to low patient numbers in each category.

Thank you for your insightful comment. Our study aims to address multiple facets of pelvic fragility fracture diagnosis and treatment, not to establish the superiority of one surgical method over another. Because of the multi-factorial problem in this patients category (diagnosis, whom to operate and how to operate) we choose for this randomised guideline implementation study. In this way a number of answers can be provided to this complex problem, but we agree that analysing the effectiveness of specific fixation techniques will be challenging due to low patient numbers in each category. This should indeed be the focus of future, more targeted studies or in our sub-analysis. In our study protocol the following advice on surgical treatment is provided to the surgeons: treatment should be as minimal invasive as possible (in line with the reviewers own advice and of several literature reviews). In both participating hospitals patients with a fragility fracture of the pelvis are almost always treated by percutaneous screw fixation (7.3) in all corridors if technical feasible (e.g. antegrade/retrograde rami screws/LC2/TITS and if technical feasible by multiple TITS).

Page 16/30, line 58: 60 weeks???

I have changed the number of participating centres to the correct number (9), and have specified that the total number of weeks of the study duration is 66 weeks. Thank you.

References

1. Mennen, A.H.M., et al., Pelvic Ring Fractures in Older Adult Patients-Assessing Physician Practice Variation among (Orthopedic) Trauma Surgeons. J Clin Med, 2023. 12(19).

VERSION 2 – REVIEW

**REVIEWER NAME**	Rommens, Pol Maria
**REVIEWER AFFILIATION**	University Medical Center of the Johannes Gutenberg University Mainz
**REVIEWER CONFLICT OF INTEREST**	None
**DATE REVIEW RETURNED**	08-Jul-2024

**GENERAL COMMENTS**	All comments and requests have been answered adequately .
